# Prevalence and Influence of Genetic Variants on Follow-Up Results in Patients Surviving Thoracic Aortic Therapy

**DOI:** 10.3390/jcm13175254

**Published:** 2024-09-05

**Authors:** Tamer Ghazy, Nesma Elzanaty, Helmut Karl Lackner, Marc Irqsusi, Ardawan J. Rastan, Christian-Alexander Behrendt, Adrian Mahlmann

**Affiliations:** 1 Department of Cardiac and Thoracic-Vascular Surgery, University Hospital Giessen and Marburg, Philipps University of Marburg, 35043 Marburg, Germany; irqsusi@med.uni-marburg.de (M.I.); a.rastan@uk-gm.de (A.J.R.); 2 Department of Medical Physiology, Tanta Faculty of Medicine, Tanta University, Tanta 31527, Egypt; nesma.elzanaty@med.tanta.edu.eg; 3 Division of Physiology, Otto Loewi Research Center for Vascular Biology, Immunology and Inflammation, Medical University of Graz, 8010 Graz, Austria; helmut.lackner@medunigraz.at; 4 Department of Vascular and Endovascular Surgery, Asklepios Clinic Wandsbek, Asklepios Medical School, 20043 Hamburg, Germany; behrendt@hamburg.de; 5 Brandenburg Medical School Theodor Fontane, 16816 Neuruppin, Germany; 6 Centre for Vascular Medicine, Clinic of Angiology, St.-Josefs-Hospital, Katholische Krankenhaus Hagen gem. GmbH, 58099 Hagen, Germany; mahlmanna@kkh-hagen.de; 7 Department of Internal Medicine III, University Hospital Carl Gustav Carus at Technische Universität Dresden, 01307 Dresden, Germany

**Keywords:** aortic surgery, aortic disease, genetic testing, heritable aortic disease

## Abstract

**Background/Objective:** To investigate the prevalence and effects of genetic variants (GVs) in survivors of thoracic aortic dissection/aneurysm repair. **Methods:** Patients aged 18–80 years who survived follow-up after cardiosurgical or endovascular repair of thoracic aortic aneurysm or dissection at a single tertiary center between 2008 and 2019 and underwent genetic testing were enrolled. The exclusion criteria were age >60 years, no offspring, and inflammatory- or trauma-related pathogenesis. Follow-up entailed computed tomography-angiography at 3 and 9 months and annually thereafter. All patients underwent genetic analyses of nine genes using next-generation sequencing. In cases of specific suspicion, the analysis was expanded to include 32 genes. **Results:** The study included 95 patients. The follow-up period was 3 ± 2.5 years. GVs were detected in 40% of patients. Correlation analysis according to primary diagnosis showed no significant correlation in disease persistence, progression, or in reintervention rates in aneurysm patients and a correlation of disease persistence with genetic variants according to variant class in dissection patients (*p* = 0.037). Correlation analysis according to follow-up CD finding revealed that patients with detected dissection, irrespective of original pathology, showed a strong correlation with genetic variants regarding disease progression and reintervention rates (*p* = 0.012 and *p* = 0.047, respectively). **Conclusions:** The prevalence of VUS is high in patients with aortic pathology. In patients with dissected aorta in the follow-up, irrespective of original pathology, genetic variants correlate with higher reintervention rates, warranting extended-spectrum genetic testing. The role of VUS may be greater than is currently known.

## 1. Introduction

Diseases of the thoracic aorta are often first diagnosed in the context of acute events, which frequently require urgent or emergent intervention. The disease course depends on the underlying aortic pathology and associated complications [1]. Known cardiovascular risk factors that predispose toward the development of thoracic aortic diseases over the course of life and increasing age contribute to the development of atherosclerosis, a potential pathophysiological cause of thoracic aortic pathologies. Moreover, traumatic or inflammatory influences can occasionally act as causative factors [2,3]. Conversely, diseases of the connective tissue of the aortic wall, such as Marfan syndrome or Ehlers-Danlos syndrome, which may be associated with thoracic aortic diseases, are more prevalent in younger patients [2]. Modern sequencing analyses allow for the targeted analysis of genetic loci associated with mutations linked to connective tissue diseases of the aortic wall, which can culminate in thoracic diseases of the aortic wall [2]. In addition to previously described and presumed disease-causing gene mutations, unclassified variants of unclear clinical significance (VUS) have been increasingly detected [4,5,6,7,8,9,10]. Furthermore, previous studies have focused on detecting and analyzing genetic changes that may be involved in the etiology of thoracic aortic diseases. However, the presence of VUS and their differential effects on the disease course, recurrence, and the type and number of subsequent surgical or endovascular interventions that may be needed based on these VUS have received very little attention. The primary aim of this study was to investigate the effect of genetic variants on the disease course in patients who underwent surgical or endovascular repair for thoracic aortic dissection or aneurysm. These findings are significant, not only for the patients themselves in structuring follow-up and selecting different therapeutic methods in the case of necessary reinterventions, but also potentially for future generations in the case of inheritable genetic aortic disease, enabling recommendations for screening examinations.

## 2. Materials and Method

The participating clinics/centers and collaborators included the University Vascular Center Dresden, Angiology Division, Medical Clinic and Polyclinic III, University Hospital Carl Gustav Carus, Dresden University of Technology; Heart Center, Dresden University Hospital, Department of Cardiac Surgery; and Community Practice for Human Genetics, Dresden, Germany (existing cooperation agreement with University Hospital Carl Gustav Carus).

The study was approved by the Ethics Committee of the Dresden University of Technology (EK317082014). Clinical data were obtained from the university aortic board, for which patient consent was waived. All participants provided written consent for inclusion in genetic testing.

### 2.1. Inclusion and Exclusion Criteria

All patients who underwent cardiosurgical or endovascular repair of thoracic aortic aneurysm (TAA) or thoracic aortic aneurysm and dissection (TAAD) at a single tertiary center between January 2008 and June 2019 were screened for eligibility. Screening was performed according to the primary diagnoses and treatments documented in the institutional database using the International Classification of Diseases codes. 

The inclusion criteria were as follows:

- Patient age between 18 and 80 years;
- Survival during the procedure and follow-up period;
- Patient consent to undergo human genetic testing to clarify the pathogenesis of the underlying aortic wall disease.

The exclusion criteria were as follows:

- Confirmed inflammatory or trauma-related pathogenesis of thoracic aortic disease
- Missing follow-up data;
- Failure to provide consent.

In compliance with German health insurance regulations, patients aged above 60 years without any offspring were excluded from the study.

### 2.2. Recruited Patients/Sample Size

**Figure 1:**
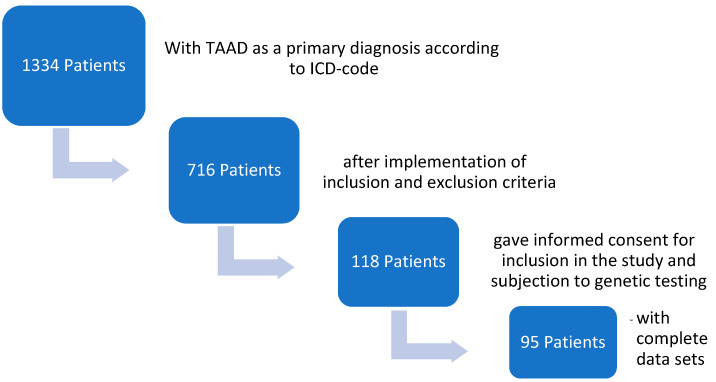
Selection criteria and patient recruitment.

The primary diagnosis and treatment of the thoracic aortic pathology of 1334 patients were reviewed, and their primary data and patient records were screened. A total of 716 patients met the eligibility criteria. After contact, 118 patients consented to undergo genetic testing. Follow-up and human genetic data were missing for 23 patients; finally, 95 patients were enrolled in the study, as seen in Figure 1.

### 2.3. Structured Follow-Up Examinations after Surgical or Endovascular Treatment of Aortic Pathology

Patients were subjected to the following structured clinical and image-based follow-up at the study institution after surgical treatment or thoracic endovascular aortic repair (TEVAR) for thoracic aortic pathology. During follow-up, the patient’s medical history, current medication, cardiovascular risk factors, and comorbidities were recorded. Routine follow-up examinations were typically conducted 3 and 9 months after the primary intervention, and annually thereafter. Unscheduled assessments were performed earlier, if indicated, which was determined by the interdisciplinary vascular conference (aortic board) comprising experts from cardiothoracic surgery, vascular surgery, interventional radiology, angiology, and cardiology.

If there were no contraindications, such as severely impaired renal function, clinical hyperthyroidism, or iodine allergy, computed tomography (CT) angiography was preferred at the 3-month and 1-year follow-ups. In cases with a stable course of aortic disease without the need for treatment, magnetic resonance angiography was performed annually, alternating with transesophageal echocardiography combined with abdominal aortic ultrasonography.

### 2.4. Human Genetic Analysis to Clarify the Pathogenesis of Aortic Disease

Next-generation sequencing (NGS) was performed after DNA extraction from the blood samples collected from the patients. Initially, capture-based enrichment was performed, followed by analysis using the MiSeq Desktop Sequencer© (Illumina, San Diego, CA, USA). For the genes listed in Table 1, biometric data evaluation, including copy number variation (CNV) analysis, was conducted to identify larger deletions or duplications. Areas that could not be adequately assessed were further analyzed using conventional Sanger sequencing or multiplex ligation-dependent probe amplification. The identified polymorphisms, which are considered non-pathogenic according to current knowledge, have been reported separately. A five-tier classification was applied based on the methodology of Plon et al. and the Yale Aortic Institute Team [10,11]:

1. Class 1: Not disease-causing/nonpathogenic or of no clinical relevance;
2. Class 2: probably not disease-causing or of minor clinical relevance;
3. Class 3: VUS;
4. Class 4: likely disease-causing and pathogenic;
5. Class 5: disease-causing/pathogenic.

Table 1 shows the gene loci studied in the cohort, which were classified into classes 4 and 5 based on Plon et al.’s [11] study.

Initially, the nine genes assumed to most commonly harbor disease-causing variants were examined. In cases of specific suspicion, such as familial aggregation, the analysis was expanded to a total of 32 genes, covering the currently known genetic causes of non-syndromic TAAD to a large extent, after obtaining consent again from the patient. If indications of a syndromic form were present, additional investigations such as chromosome analysis for suspected Turner Syndrome were initiated.

### 2.5. Applied Statistical Methods

Continuous variables were presented as the mean ± standard deviation and binary data as a percentage of the population. Pearson’s chi-squared and F-tests were used to examine the correlation between distinct binary traits. As cardiovascular risk factors were not metrically scaled, Kendall’s tau was used for the calculation. Statistical significance was set at *p* < 0.5 (two-sided). SPSS Statistics software (version 27, IBM, New York, NY, USA) was used for the statistical analysis.

## 3. Results

### 3.1. Demographic and Preoperative Data

The study population included 95 patients with a preponderance of males (71% men vs. 29% women). There was no significant difference in the age distribution at initial diagnosis, during human genetic examination, and on the cutoff date of 30 June 2019 (defined as the end of data collection) (Table 2 and Table 3).

Patients who underwent surgical treatment were older than those who underwent endovascular therapy (59.4 ± 12.0 vs. 48.0 ± 10.5 years, respectively, *p* < 0.001); the frequency of men was higher in the surgical treatment group (*p* = 0.044).

Irrespective of sex, arterial hypertension was the dominant preexisting cardiovascular risk factor, as seen in 83% of patients, followed by a history of smoking and hypercholesterolemia (34 and 33%, respectively) and coronary heart disease (14%). Carotid stenosis/stroke, peripheral arterial occlusive disease, chronic obstructive pulmonary disease, or renal insufficiency were present in <10% of patients (Table 4).

Nearly all the patients were on long-term medication; 95% of patients were receiving beta-blocker therapy, and 83% were on angiotensin-converting enzyme inhibitors or angiotensin II type 1 receptor antagonists. Other antihypertensive agents were used in 21% of patients. Statin therapy was documented in 33% of patients. Antiplatelet therapy was administered to 43% of patients. Oral anticoagulants were administered to 37% of the study cohort (Table 5); the indications for therapeutic anticoagulation included mechanical heart valve replacement, atrial fibrillation/flutter, or past history of venous thromboembolism (Table 5).

Overall, 57% of patients had thoracic aortic dissection, and 41% of patients had thoracic aneurysm. At the time of the initial diagnosis, 2% of patients exhibited a concomitance of both conditions. Contained rupture of the aortic aneurysm was confirmed radiologically in 6.3% of patients. Intramural hematoma and penetrating aortic ulcer were observed in 8.4% and 2.1% of patients, respectively.

### 3.2. Operative Data

Surgery constituted the primary treatment for thoracic aortic pathologies in 68% of this patient population, while an endovascular interventional approach was employed in the remaining 32%. The main aortic diagnosis (thoracic aortic dissection vs. aneurysm) and the type of treatment implemented (surgical vs. endovascular) did not differ significantly in this cohort (Pearson Chi-Square, χ^2^ = 0.278, *p* = 0.598). The aortic procedures are summarized in Table 6.

### 3.3. Follow-Up Data

#### 3.3.1. Data Analysis According to Primary Diagnosis

##### General Results

The average follow-up duration was about 3 ± 2.5 years. Imaging studies showed complete resolution of the existing aortic pathology after the primary procedure in 31% of patients. The distribution of residual pathology is shown in Table 7.

The correlation analysis showed no significant correlation of genetic variants to the persistence of pathology (*p =* 0.414), diameter expansion, development of new aneurysms (*p =* 0.369), or reinterventional rate (*p =* 0.742). The analysis showed a significant correlation with the development of new dissections or the expansion of dissection to previously non-dissected segments (*p* = 0.042).

During follow-up, 38% of patients required one or more reinterventions; a maximum of four additional surgical or endovascular procedures were performed during follow-up. There was no significant correlation with genetic variant detection (*p* = 0.742).

##### Aortic Aneurysm Patients

Secondary expansion of the aortic diameter or de novo development of aortic ectasia/aneurysm occurred in 23 patients. Six patients with an initial aortic aneurysm subsequently developed aortic dissection in non-treated segments during follow-up. No retrograde aortic dissection was observed after TEVAR. 

In patients who were primarily treated for aortic aneurysm and developed new dissections in the subsequent follow-up, the prevalence of genetic variants was 80%, and that of VUS was 60%. In patients who developed a de novo aneurysm or further expansion of an existing aneurysm in distant segments, the prevalence of genetic variants was 50%, and that of VUS was 37.5%. However, there were no statistically significant differences in the prevalence compared to the stable cohort (*p* = 0.405 and *p* = 0.633 respectively). Supplementary Table S1 lists the detected genetic variants in aneurysm patients and the state of disease progression in each patient with a genetic variant. The analysis of reintervention rates showed no significant correlation with genetic variant detection (*p* = 0.248).

##### Aortic Dissection Patients

Table 8 presents the trends in thrombosis of the false lumen over time after the primary procedure for all treated dissections. Overall, 65.6% of the cohort retained a completely or partially perfused false lumen after aortic dissection during follow-up, whereas 32.3% showed complete thrombosis immediately after the primary procedure or during follow-up imaging.

The correlation analysis of persistent pathology with genetic variants shows a borderline significance (*p* = 0.05, Table 9) when all variants are evaluated as one pool. 

The correlation analysis of persistent pathology with genetic variants according to variant class shows a significant correlation (*p* = 0.037, Table 10).

In the event of residual aortic dissection after the primary dissection procedure, 85% of patients followed a stable course during subsequent follow-ups. In 15% of patients with residual dissections, progression with extension into the initially unaffected vessel sections was detected. The prevalence of genetic variants was 24%, and that of VUS was 22% in patients with a stable disease course. The prevalence of genetic variants was 42% in patients with disease progression, all of which were VUS. There was no significant correlation between disease progression and genetic variants (*p =* 0.426). Supplementary Table S2 enumerates the genetic variants detected in patients with dissection progression. The analysis of reintervention rates showed no significant correlation with genetic variant detection (*p* = 0.250).

#### 3.3.2. Data Analysis According to Follow-Up CT-Finding

The evaluation of all patients with detected aortic dissection at follow-up (i.e., aneurysm patients who show a new dissection in the follow-up and dissection patients who show a persistent dissection in follow-up) showed a strong correlation with the presence of genetic variants (Spearman R^2^ = 0.321, *p* = 0.012; Kendall Tau R^2^ = 0.321, *p* = 0.019).

The analysis of reintervention rates showed a positive correlation in reintervention rates in patients with detected variants (Spearman R^2^ = 0.256, *p* = 0.046; Kendall Tau R^2^ = 0.243, *p* = 0.047).

### 3.4. Human Genetic Analysis: Gene Variants Distribution in the Studied Cohort

Human genetic analysis revealed variants in the target candidate genes with a known connection to connective tissue diseases involving the aortic vessel wall in 40.7% of patients (n = 38, Table 11). Seven patients (7.4% of the total cohort, 18.4% of all variants) harbored a class 5 variant with an established association with connective tissue diseases [8].

Upon strict segregation of cases with a singular occurrence of a class 5 pathological variant, a lower-class variant, or a combination thereof, the following frequencies emerged: Thirty-one patients harbored a lower-class variant, accounting for 81.6% of all detected variants; four patients (10.5%) exclusively harbored a pathological class 5 variant; in three patients (7.9%), both a pathological variant and a lower-class variant were found. Table 11 details the affected genes and variant classes. The analysis discovered no difference in the detected variants with respect to sex (Pearson’s Chi-Square: χ^2^ = 0.684; *p* = 0.408).

## 4. Discussion

Aortic aneurysms or dissections can remain asymptomatic for a long time but can emerge suddenly, causing acute life-threatening events. Despite intensive research and the accumulation of extensive data, definitive identification of the individual pathophysiological causes of aortic vascular wall pathologies is not often possible. In addition to a likely combination of various cardiovascular risk factors such as arterial hypertension, hyperlipoproteinemia, diabetes mellitus, or smoking, the familial form, including connective tissue diseases, is the second most common cause [6,7]. In syndromic connective tissue diseases such as Marfan syndrome or Ehlers-Danlos syndrome, screening for aortic manifestations using imaging techniques has been established because of the known association with aortic involvement [12,13]. 

Rapid advancements in the field of genetic diagnostics have fundamentally altered the diagnosis and investigation of many disease patterns. The ability to completely analyze the human genome is particularly significant for identifying the causes of familial clustering. Specific deviations from the norm, i.e., the wild-type, can be clinically and pathophysiologically correlated with the pathological findings and can be classified as pathological, allowing screening and early diagnostics to be offered to the relatives of affected individuals. The frequency of detection of previously unclassified mutation variants is on the rise, which must be evaluated in terms of their clinical significance and the pathogenesis of aortic diseases.

In recent years, numerous scientific studies have undertaken a search for genetic triggers of thoracic aortic diseases such as aneurysms or dissections [5,7,10,11,14]. In addition to the importance of known mutations, such as changes in the fibrillin 1 gene (FBN1) in Marfan syndrome, which have already been described in association with connective tissue diseases [8,15], other genes have also been recognized as relevant to the disease-causing process and investigated in greater depth, expanding knowledge about the variety of possible genetic triggers [4,9,16,17,18,19].

In the presence of genetic aortopathy, depending on the type and severity of the aortic connective tissue disease, treatable secondary aortic pathologies may develop over time, even after surgical or endovascular treatment of the thoracic aneurysm or dissection, with varying dynamics. Currently, data are insufficient on the influence of genetic variants on the development of these secondary aortic manifestations, with potential indications for re-treatment/reintervention in patients with gene mutations/mutation variants related to aortic connective tissue diseases.

Many studies have focused on the prevalence of changes in the genotype as the pathogenic basis for TAAD, yielding results similar to those of the current study. Poninska et al. analyzed 10 known disease-associated genes in 51 patients with TAAD and found mutations in 41.2% of participants, with 35.3% being classified as LP or pathogenic disease-causing variants. The inclusion of patients was primarily but not exclusively based on the presence of familial clustering, early age of onset, and suspected connective tissue disease [14]. Fang et al. investigated 11 causal genes in 70 patients with TAAD from southern China using NGS and found deviations from the wild-type in 51.4% of patients. Of these, 7.5% were definitively pathological, 25% were likely pathological variants, and 32.5% were potentially disease-causing mutations that closely matched the results of the present study. Familial clustering was not a prerequisite for inclusion, but if present, the variant detection rate was 92.3% compared to 45.6% in participants without familial clustering [20]. Ziganshin et al. included 102 patients with TAAD, familial clustering, and early age of onset, who were examined using whole-exome sequencing for 21 genes. Here, variants were described in 27.5% of all tested patients, with 21.6% in the VUS category [21]. In a study conducted in northwest China by Li et al., 51.4% of 212 patients with TAAD tested positive for mutations using NGS. A total of 31.6% fell into the LP and definitely pathological variant group and 19.8% into VUS. The working group tested 15 genes associated with TAAD [22]. An even larger genetic sample was studied by Li et al. in southern China, who searched for mutations in 129 genes in 151 patients with sporadic or familial TAAD and described abnormalities in 62.3% of all cases. The majority of these genotypic changes fell into the VUS group, and 22.5% included likely and definitely pathological deviations [23].

Nonetheless, the Yale Aortic Institute team examined a cohort of 967 participants with and without familial clustering using NGS for 15 TAAD-associated genes and described VUS, LP, and pathogenic variants in only 12% of the participants. Forty-nine cases of pathological or likely pathological variants were identified, accounting for 4.9%. It is noteworthy that approximately half of these participants had no relevant family history [10]. 

All the above-mentioned studies, as well as the present one, concur that disease-associated changes occurred most frequently in the FBN1 gene. The frequencies of variants in MYH11, MYLK, NOTCH1, ACTA2, COL1A1/2, COL3A1, and COL5A1/2 were not entirely uniform [5,10,20,21,23,24]. Further investigation of other gene loci and their pathogenic associations with TAAD is required, especially in non-syndromic diseases with familial clustering [24,25].

To date, mutations in 37 gene loci with a pathophysiological association with thoracic aortic aneurysms and dissections have been detected [5]. Li et al. clearly showed that research on other causal genes (with 129 detected gene loci) is rapidly progressing and will continue to do so [23]. The frequency of detected variants varies greatly among studies, ranging from 12% to 62.3% without further selection or from 4.9% to 35.3% when considering only likely and definite pathological variants. The large variation in prevalence in these studies can be attributed to different study designs with a narrower/broader selection of patients and expansion of the gene loci under examination [5,10,14,20,21,23,24]. Apropos of the variant prevalence in the current study, especially considering the sample size and the number of analyzed gene loci, the results align with existing evidence. Nevertheless, closer examination of these studies may pave the way for more refined indications for genetic testing. At this juncture, we believe that genetic testing should continue to expand, as further examination of gene loci, specific mutations, and their prevalence and clinical relevance is needed to determine clearer pathways for future decision-making.

Regarding a possible connection/influence of a confirmed genetic variant and the rate of required reinterventions during follow-up, the present study found no significant correlation with the primary diagnosis. Interestingly, there was a statistically significant increase in the risk of reintervention for patients with detected aortic dissection in the follow-up with a genetic variant. This is consistent with the results of Poninska et al., who described a shorter event-free interval for patients with pathological and likely pathological mutations in their study [14]. This may indicate a closer, more meticulous follow-up in this cohort, which may lead in the future to a lower threshold for intervention/reintervention. 

Nevertheless, these consequences are dependent on the confirmation/exclusion of genetic variants in patients with dissection, which is not always logistically possible as genetic-variant analysis using NGS is currently only approved for TAAD with familial clustering or conspicuous age or phenotype. This strategy should be parsed critically from an economic perspective. Performing human genetic-variant testing to clarify the pathogenesis of aortic disease is valuable, as it may have clinical relevance for the patient, such as admission to a structured follow-up in specialized centers to ensure timely detection of dynamic aortic changes and reinterventions (if required), or for his/her offspring to facilitate early detection of potentially at-risk offspring and offer genetic testing, imaging screening, and follow-up. These benefits also offer sound economic arguments for prophylactic medicine, and the economic advantages should be studied and included in the decision-making processes. Currently, from an economic perspective, the question of a possible age limit or cutoff value from which genetic testing is no longer meaningful arises, especially since at an older age, the first manifestation of aortic diseases is more likely to be caused by atherosclerosis than by a connective tissue disease based on genetic variants. Although the data from our study show that patients with the first manifestation of a thoracic aortic dissection or aneurysm disease and a confirmed genetic variant were, on average, about six years younger, this trend did not attain significance in the analysis by age group, and the prevalence of genetic variants did not significantly differ according to the age group, indicating that genetic testing can be beneficial to older patients. 

**Figure 2:**
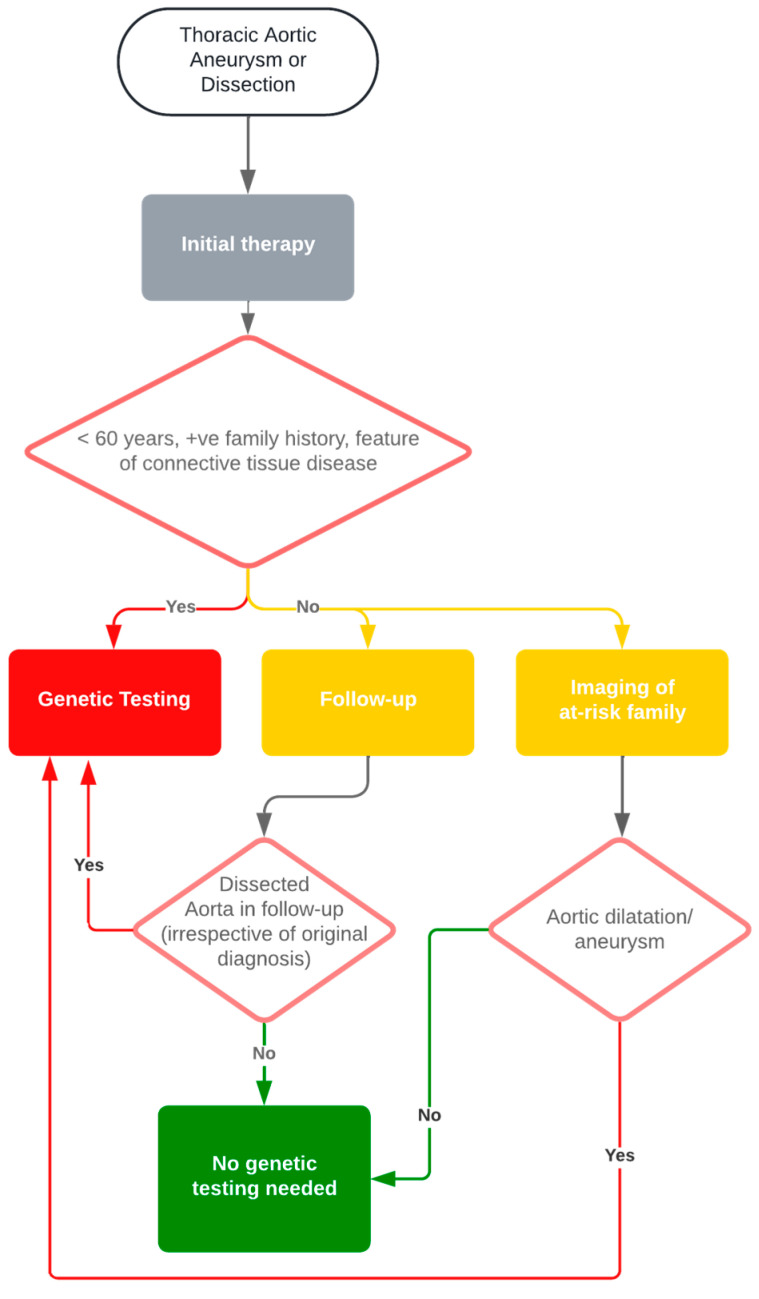
Decision-making flowchart for genetic testing of patients with TAAD.

The first step in this pathway was recommended by Caruana et al., who recommended genetic analysis irrespective of age in the absence of risk factors and pre-existing conditions, but with an absolute cutoff of 70 years [26]. Even though a cutoff to determine the age limit of human genetic testing would be desirable, it cannot yet be conclusively determined owing to the current paucity of data. Our results do not show a clinical benefit for patients with aortic aneurysm, but based on the significantly higher percentage of reintervention in patients with detected dissection in the follow-up with genetic variants in our study, we suggest the following flowchart for decision-making regarding genetic testing in TAAD patients according to our results (Figure 2).

Our data and suggestions might be a step on a long way to gathering evidence on a topic that lacks supporting data. This is confirmed in the last guidelines of the European Association of Cardiothoracic Surgery for diagnosing and treating acute and chronic syndromes of the aortic organ [27], which confirm the lack of evidence-based standardized follow-up protocol specific to each disease and treatment modality. Although variants of specific genes are now included in the guidelines, with a lower threshold for indicating therapy, large nationwide or multinational cohort studies are urgently needed to expand the knowledge on genetic variants affecting the aortic disease and its course. 

### Limitations

The study limitations include retrospective enrolment of patients with a subsequent inevitable lower recruitment percentage, which would have affected the generalizability of the study results. However, our group eliminated selection bias in an earlier publication with the same patient cohort [28]. Furthermore, the results concur with those of most previous studies. The genetic analysis included a specific group of genes as approved by the German health authorities. Expanding the spectrum of genetic testing might provide further insights, which our study group intends to perform in future research. Furthermore, it should be noted that mosaicism or other functionally significant genetic alterations in the examined genes or influencing regions cannot be entirely excluded. The study was performed at a single center, which might have influenced or limited the geographical area from which the patient sample was drawn. The influence of surgeons/surgery type on reintervention was not investigated.

## 5. Conclusions

The prevalence of genetic variants is high in patients with aortic pathology, a large percentage of which are currently classified as VUS. In patients with dissected aorta in the follow-up, irrespective of original pathology, the detected genetic variants correlated with higher reintervention rates, which indicates that VUS may, in fact, play a larger role than is currently known. Studies with larger cohorts are needed to further examine the detected variants to confirm their correlation and change their class to pathological or probably pathological.

## Figures and Tables

**Table 1:** Investigated gene loci associated with syndromic or non-syndromic thoracic aortic diseases with aneurysm and/or dissection.

Gene Name	Protein	Associated Aortic Disease/Syndrome
ACTA2 (NM_001613.2)	Smooth muscle α-actin	TAAD, AAT6 multisystem smooth muscle dysfunction, MYMY5
AEBP1 (NM_001129.4)	AE binding protein 1	BAA, EDS
BGN (NM_01711.5)	Biglycan	TAAD, Meester-Loeys Syndrome
COL1A1 (NM_000088.3)	Collagen type I α1 chain	TAAD, EDS
COL3A1 (NM_000090.3)	Collagen type III α1 chain	TAAD, EDS vascular Type (IV)
COL4A5 (NM_000495.3)	Collagen type IV α5 chain	TAAD, Alport Syndrome (Collagen type IV deficiency)
COL5A1 (NM_000093.4)	Collagen type V α1 chain	TAAD, EDS classical Type I
COL5A2 (NM_000393.4)	Collagen type V α2 chain	TAAD, EDS classical Type II
EFEMP2 (FBLN4)(NM_016938.4)	Fibulin-4	TAAD, other arterial aneurysms, Cutis laxa (autosomal recessive) Type Ib
ELN (NM_000501.3, NM_001278939.1)	Elastin	TAAD, Cutis laxa (autosomal dominant)
FBLN5 (NM_006329.3)	Fibulin 5	TAAD, Cutis laxa, Macular degeneration
FBN1 (NM_000138.4)	Fibrillin-1	TAAD, AAA, other arterial aneurysms, Marfan Syndrome
FBN2 (NM_001999.3)	Fibrillin-2	TAAD, Congenital Contractural Arachnodactyly
FLNA (NM_001110556.2)	Filamin A	TAAD, Periventricular nodular heterotopia, Otopalatodigital Syndromes
FOXE3 (NM_012186.2)	Forkhead box E3	TAAD, AAT11
GATA5 (NM_080473.4)	GATA binding protein 5	TAAD
LOX (NM_002317.6)	Lysyl oxidase	AAD, AAA, AAT10
MAT2A (NM_005911.5)	Methionine adenosyltransferase II α	TAA, FTAA
MFAP5 (NM_003480.3)	Microfibril-associated glycoprotein 2	TAAD, AAT9
MYH11 (NM_002474.2)	Smooth muscle myosin heavy chain	TAAD, AAT4
MYLK (NM_053025.3)	Myosin light chain kinase	TAAD, AAT7
NOTCH1 (NM_017617.4)	Notch receptor 1	TAAD, AOVD
NOTCH3 (NM_000435)	Notch receptor 3	TAAD
PLOD1 (NM_000302.3)	Procollagen-lysine,2-oxoglutarate 5-dioxygenase 1	TAAD, EDS
PRKG1 (NM_006258.3)	Type I cGMP-dependent protein kinase	TAAD, AAA, AAT8
RLP26 (NM_000987.4)	Receptor-like protein 26	TAAD
SKI (NM_003036.3)	Sloan Kettering proto-oncoprotein	TAA, Shprintzen-Goldberg Syndrome
SLC2A10 (NM_030777.3)	Glucose transporter 10	TAA, other arterial aneurysm

AAT (n): familial thoracic aortic aneurysms (1–11) TAA: thoracic aortic aneurysm, TAAD: thoracic aortic aneurysm and dissection, EDS: Ehlers-Danlos syndrome, BAA: abdominal artery aneurysm; AOVD: aortic valve disease.

**Table 2:** Age distribution of the patient population.

Patients	Women (n = 28)	Men (n = 67)	*p* (*t*-Test)
Age at diagnosis	52.4 ± 12.4	51.3 ± 12.2	0.696
Age at genetic testing	55.3 ± 12.6	53.8 ± 12.5	0.596
Age as of 30 June 2019	55.8 ± 12.2 years	54.5 ± 12.0 years	0.612

**Table 3:** Primary treatment for the main diagnosis of thoracic aortic dissection/aneurysm based on age and sex.

Patients	Primarily Treated with Endovascular Therapy (n = 30)	Primarily Treated with Open Cardiosurgery (n = 65)	*p*-Value
Age (in years) at genetic testing	59.4 ± 12.0	48.0 ± 10.5	<0.001 (*t*-test)
Age as of 30 June 2019	63.7 ± 11.5	50.8 ± 10.0	<0.001 (*t*-test)
Sex (n) (women/men)	13/17	15/50	0.044 (Chi-Squared Test)

**Table 4:** Comorbidities and cardiovascular risk factors in the patient population.

Cardiovascular Risk Factors	n	Percentage
Arterial Hypertension	79	83%
(Ex) Smoking	32	34%
Hypercholesterolemia	31	33%
Diabetes Mellitus	8	8%
Coronary Heart Disease	13	14%
**Comorbidities**		
Renal Insufficiency	9	9%
Carotid Stenosis/Stroke	5	5%
Peripheral Arterial Occlusive Disease (PAOD)	4	4%
COPD	2	2%

COPD: chronic obstructive pulmonary disease.

**Table 5:** Long-term medication of the patient population.

Medication	n	Percentage
Beta-Blockers	90	95%
ACE Inhibitors/AT1 Receptor Antagonists	79	83%
Platelet Aggregation Inhibitors	40	42%
Calcium Antagonists	39	41%
Oral Anticoagulants	35	37%
Statins	31	33%
Other Antihypertensives	20	21%

ACE: angiotensin-converting enzyme, AT1: angiotensin II type 1.

**Table 6:** Management of the primary diagnosis.

Procedure	Frequency
Isolated replacement of the ascending aorta	22%
Aortic root replacement with/out valve sparing, with hemiarch replacement	32%
Aortic arch replacement	3%
(Frozen) Elephant trunk operation (FET)	7%
TEVAR (all)	25%
TEVAR with complete stenting of left subclavian artery	11%
TEVAR with partial stenting of left subclavian artery	8%
With creation of carotid-carotid bypass	2%
With creation of carotid-subclavian bypass	0%
TEVAR octopus debranching and EVAR	1%

EVAR: endovascular aortic aneurysm repair, TEVAR: thoracic endovascular aortic aneurysm repair.

**Table 7:** Distribution of residual aortic pathology.

	Type of Persistent Pathology			Total
	Resolved Pathology	Residual Aneurysm	Residual Dissection	
Resolved Pathology	28	0	0	28
Residual pathology	1	9	57	67
Total	29	9	57	95

**Table 8:** Aortic dissections: Dynamics of false lumen thrombosis at follow-up.

Trend in False Lumen	Frequency
Not perfused/resolved immediately postoperatively	26.9%
Completely thrombosed at follow-up	54%
Partial thrombosis stable	18.3%
Progressive thrombosis	9.7%
Completely perfused	37.6%

**Table 9:** Correlation of persistence of dissection to genetic variants.

	Presence of Genetic Variants			
	Non-Present	Present		
Resolution of aortic pathology	2	4	6	
Residual dissection	35	13	48	
	37	17	54	*p =* 0.05

**Table 10:** Correlation of persistence of dissection to genetic variant classes.

	Presence of Genetic Variant Class				
	Non-Present	Class 4 or 5	Lower Classes		
Resolution of aortic pathology	2	0	4	6	
Residual dissection	35	2	11	48	
	37	2	15	54	*p =* 0.037

**Table 11:** Overview of the results of variant analysis.

Affected Gene	Variant Class 4 or 5	Absolute Number of Patients	Percentage of Cohort
FBN1	yes	3	7.9%
	no	10	26%
COL3A1	yes	1	2.6%
SMAD3	no	1	2.6%
TGFB2	yes	1	2.6%
	no	3	7.9%
TGFBR1	no	2	5.3%
MYLK	no	2	5.3%
MYH11	no	8	21%
PRKG1	no	1	2.6%
NOTCH1	no	3	7.9%
NOTCH3	yes	1	2.1%
TGRBR2	no	1	2.1%
ACTA2	no	2	5.3%
SMAD6	yes	1	2.6%

## Data Availability

The data supporting the findings of this study are available from the corresponding author upon reasonable request. The data will be available for five years after publication.

## Supplementary Materials

The following supporting information can be downloaded at: https://www.mdpi.com/article/10.3390/jcm13175254/s1, Table S1: Genetic Variants Detected in Patients with Aneurysm and Their Correlation with Disease Progression; Table S2: Genetic Variants Detected in Patients with Dissection and Their Correlation with Disease Progression.
