# Supplementary material for: Prevalence and Influence of Genetic Variants on Follow-Up Results in Patients Surviving Thoracic Aortic Therapy
Source: J Clin Med. 2024 Sep 5;13(17):5254. doi: 10.3390/jcm13175254 (PMC11396620; doi:10.3390/jcm13175254)
Supplement: Supplementary file 1 [file jcm-13-05254-s001.zip › jcm-3134559-supplementary.pdf]

Prevalence and Influence of Genetic Variants on Follow-up Results in Patients Surviving Thoracic Aortic Therapy

Supplementary data

Table S1: Genetic Variants Detected in Patients with aneurysm and Their Correlation with Disease Progression

Patient No.	Disease progression	
1	stable	heterozygote Mutation in MYLK gene, E7 c.460A>G (Ser154Gly); class 3 , unclassified variant with tendency toward pathogenicity
2	stable	Missense-Mutation in COL3A1 gene, E10 c.754G>A, Gly252Ser
3	stable	heterozygote change c.4962-1G>T, p.(?) in MYLK gene, classified as probably pathogenic (class 4)
4	progressive	heterozygote Deletion of the TGFB2 gene and neigbour gene
5	stable	heterozygote gene change in Exon 1 of the FBN1 gene (c.59A>G, p.(Tyr20Cys)), classe 3
6	stable	heterozygote sequence change c.4579-6C>T in Intron 33 of the MYH11-gene, no sign of disease relevanz
7	progressiv	pathogenic heterozygote de novo Mutation in FBN1 gene: NM_000138.4:c.4520G>A, p.(Gly1507Asp), class 5
8	progressive	in exon 57 of the FBN1 gene sequence change c.7119C>T, p.(=) heterozygous, class 3
9	stable	heterozygous duplication MYH11 gene; heterozygous variant in exon 18 of the NOTCH1 gene (c.2753A>G, p.(Asn918Ser))
10	progressive	heterozygous index mutation of exon 4 of the NOTCH3 gene (c.544C>T, p.Arg182Cys het)
11	stable	heterozygous gene change in exon 64 of the FBN1 gene (c.8176C>T, p.(Arg2726Trp)), unclassified variant with a tendency towards low clinical relevance

12	stable	heterozygous mutation in exon 6 of the FBN1 gene (c.576C>G, p.(Asn192Lys)) class 3 + heterozygous mutation in exon 53 of the FBN1 gene (c.6604A>G, p.(Met2202Val)) class 3
13	stable	heterozygous gene change in exon 1 of the FBN1 gene (c.139G>A, p.(Gly47Ser))
14	stable	heterozygous gene change in the TGFBR1 gene (c.1433A>G, p.Asn478Ser), classified as possibly causing disease (class 4)
15	stable	heterozygous variant of unclear significance in the MYH11 gene (NM_001040113.1): c.5110G>A, p.(Ala1704Thr), rs538145374), class 3
16	stable	heterozygous gene change in the TGFBR1 gene, exon 1 (c.272G>A, p.Arg91His), classified as possibly causing disease (class 4)
17	stable	heterozygous variant c5343G>A p.(Val1781=) in the FBN1 gene --> unclassified variant with a tendency towards low clinical relevance, also heterozygous gene change c.253C>G p.(His85Asp) in the SMAD3 gene --> unclassified variant with a tendency towards pathogenicity
18	stable	heterozygous gene change in exon 24 of the FBN1 gene (c.2956G>A, p.(Ala986Thr)), class 3
19	stable	in exon 34 of the MYH11 gene: gene variant c.4673C>T, p.(Thr1558Met), heterozygous, class 3
20	progressive	in exon 3 of the TGFB2 gene heterozygous variant c.548G>A, p.(Arg183His), class 3 with a tendency towards pathogenicity --> possibly LDS type 4, but not definitely confirmed

Table S2: Genetic Variants Detected in Patients with Dissection and Their Correlation with Disease Progression

Patient. No.	Dissection progression	Genetic variant
1	Stable	heterozygous FBN1 gene point mutation c.59A>G, p.Tyr20Cys, unclassified variant, unlikely to cause disease
2	Stable	on the long arm of one of the 2 chromosomes 16 gain of about 1.3 Mb of genetic material --> microduplication syndrome 16p13.11. The MYH11 gene, which lies within this region, is completely duplicated --> heterozygous duplication MYH11 gene. Array CGH result: arr[hg18]16p13.11(14876356_16199736)x3
3	Stable	Heterozygous gene change c.619G>C, p.(Val207Leu) detected in exon 3 of the TGFB2 gene, unclassified variant (class 3) with a tendency towards class 2 (rather non-pathogenic)
4	Stable	Silent Mutation Exon22 FBN1-Gen, heterozygous c.2592C>A
5	Stable	Turner syndrome --> karyotype 45,X
6	Stable	heterozygous gene change in intron 39 of the FBN1 gene (c.4943-12T>C), unclassified variant
7	Stable	PRKG1 Pathogenic Variant Substitution G>A, Exon 3, heterozygous
8	Stable	heterozygote Mutation in Exon 1 of SMAD6-gene (c.173C>G, p.(Ser58Cys)
9	Stable	heterozygous gene change (c.1289C>T, p.Pro430Leu) in exon 10 of the FBN1 gene, unclassified variant (class 3)
10	Stable	in exon 23 of the NOTCH1 gene: gene change c.3788G>A, p.(Arg1263His), heterozygous, unclassified variant with a tendency towards pathogenicity
11	Stable	ACTA2 gene, c.743A>G, p.Gln248Arg, heterozygote class 3 with a tendency toward pathogenecity
12	Stable	TGFBR2 c.-107C>T heterozygote, unclassified variant, might be pathogenic
13	Stable	in Exon 33 of FBN1 gene: gene change 4099T>C (Cys1367Arg) in heterzygote form
14	Progressive	MYH11 gene, E8, c.739C>T, p.(Arg247Cys) heterozygous; According to Plon et al. Class 3, unclassified variant with a tendency towards pathogenicity
15	Progressive	heterozygous gene variant c.1000T>A, p.(Trp334Arg) in exon 6 of the TGFB2 gene, unclassified variant with a tendency towards pathogenicity + heterozygous gene variant c.5003G>A, p.(Arg1668Lys) in exon 35 of the MYH11 gene, unclassified variant

16	Progressive	in exon 5 of the ACTA2 gene: gene change c.386T>C, p.(Phe129Ser) heterozygous, class 3 (unclassified) with a tendency towards pathogenicity
17	Progressive	heterozygote gene change in Exon 34 of MYH11 gene (c.4673C>T, p.(Thr1558Met)). Class 3
